# ShinyTPs: Curating Transformation Products from Text Mining Results

**DOI:** 10.1021/acs.estlett.3c00537

**Published:** 2023-09-29

**Authors:** Emma H. Palm, Parviel Chirsir, Jessy Krier, Paul A. Thiessen, Jian Zhang, Evan E. Bolton, Emma L. Schymanski

**Affiliations:** † Luxembourg Centre for Systems Biomedicine (LCSB), University of Luxembourg, 6 Avenue du Swing, 4367 Belvaux, Luxembourg; ‡ National Center for Biotechnology Information (NCBI), National Library of Medicine (NLM), National Institutes of Health (NIH), Bethesda, Maryland 20894, United States

**Keywords:** Transformation products, Text mining, Curation, Non-target analysis, FAIR

## Abstract

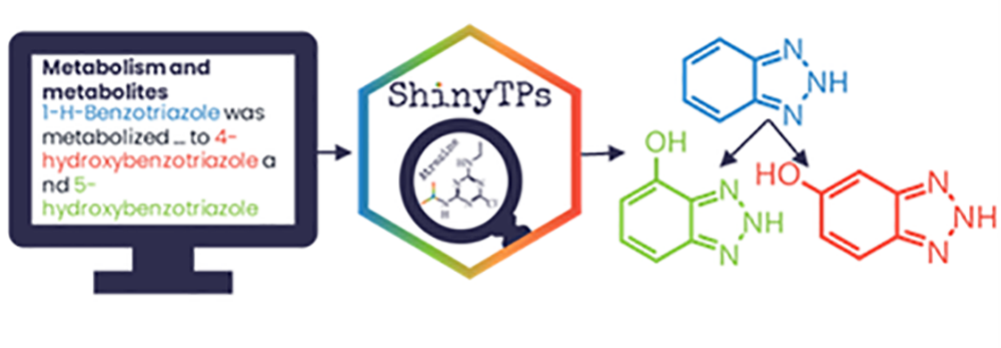

Transformation product (TP) information is essential to accurately evaluate the hazards compounds pose to human health and the environment. However, information about TPs is often limited, and existing data is often not fully Findable, Accessible, Interoperable, and Reusable (FAIR). FAIRifying existing TP knowledge is a relatively easy path toward improving access to data for identification workflows and for machine-learning-based algorithms. ShinyTPs was developed to curate existing transformation information derived from text-mined data within the PubChem database. The application (available as an R package) visualizes the text-mined chemical names to facilitate the user validation of the automatically extracted reactions. ShinyTPs was applied to a case study using 436 tentatively identified compounds to prioritize TP retrieval. This resulted in the extraction of 645 reactions (associated with 496 compounds), of which 319 were not previously available in PubChem. The curated reactions were added to the PubChem Transformations library, which was used as a TP suspect list for identification of TPs using the open-source workflow patRoon. In total, 72 compounds from the library were tentatively identified, 18% of which were curated using ShinyTPs, showing that the app can help support TP identification in non-target analysis workflows.

## Introduction

Understanding transformation product (TP) formation is crucial for assessing the compound fate and environmental risks. TPs are generally more polar and thus more mobile in the aquatic environment than their parent compounds.^1,2^ In addition, the toxicity and persistence of TPs may differ from those of their parents.^3−6^ However, there is often limited information available about TPs, both due to scarce research and inadequate FAIR^7,8^ (Findable, Accessible, Interoperable and Reusable) data on TPs, hindering utilization of and advancement on existing knowledge.

Information about TPs is provided by and available in several sources. One source of information is articles that contribute a large part of the known transformation reactions. However, the reactions are often depicted in non-machine-readable formats, such as detailed reaction schemes depicted in figures, making them difficult and time-consuming to curate and make machine-readable. Another source of transformation reaction information is databases. Some examples include MetXBioDB^9^ and SWISSPEST19^10^ as well as other NORMAN suspect lists^11^ (S66, S68 S74, S78, S79, and S81). All of these are combined within the PubChem Transformations library^12^ together with transformation reactions from ChEMBL,^13^ totalling 5923 unique reactions by CID. The Rhea^14^ database also contains balanced transformation reactions of biological interest, with over 15,000 registered reactions. These databases can be used further, including as training sets for machine learning and as suspect lists for screening for TPs.^15^ However, the number of available reactions in all databases is low in comparison to the >350,000 compounds and mixtures registered for production and use on the global market,^16^ thus showing the need to expand these resources to include more of the known transformation reactions. Looking beyond reactions, several databases also contain text-based information about transformation reactions. Examples include PharmaGKB,^17,18^ DrugBank,^19^ the Toxin and Toxin Target Database,^20^ and the Hazardous Substance Data Bank (HSDB).^22^ Several of these data sources are included within the PubChem database. These data sets often facilitate finding metabolic information for specific compounds. However, finding information for large data set purposes remains a time-consuming task.

**Figure 1:**
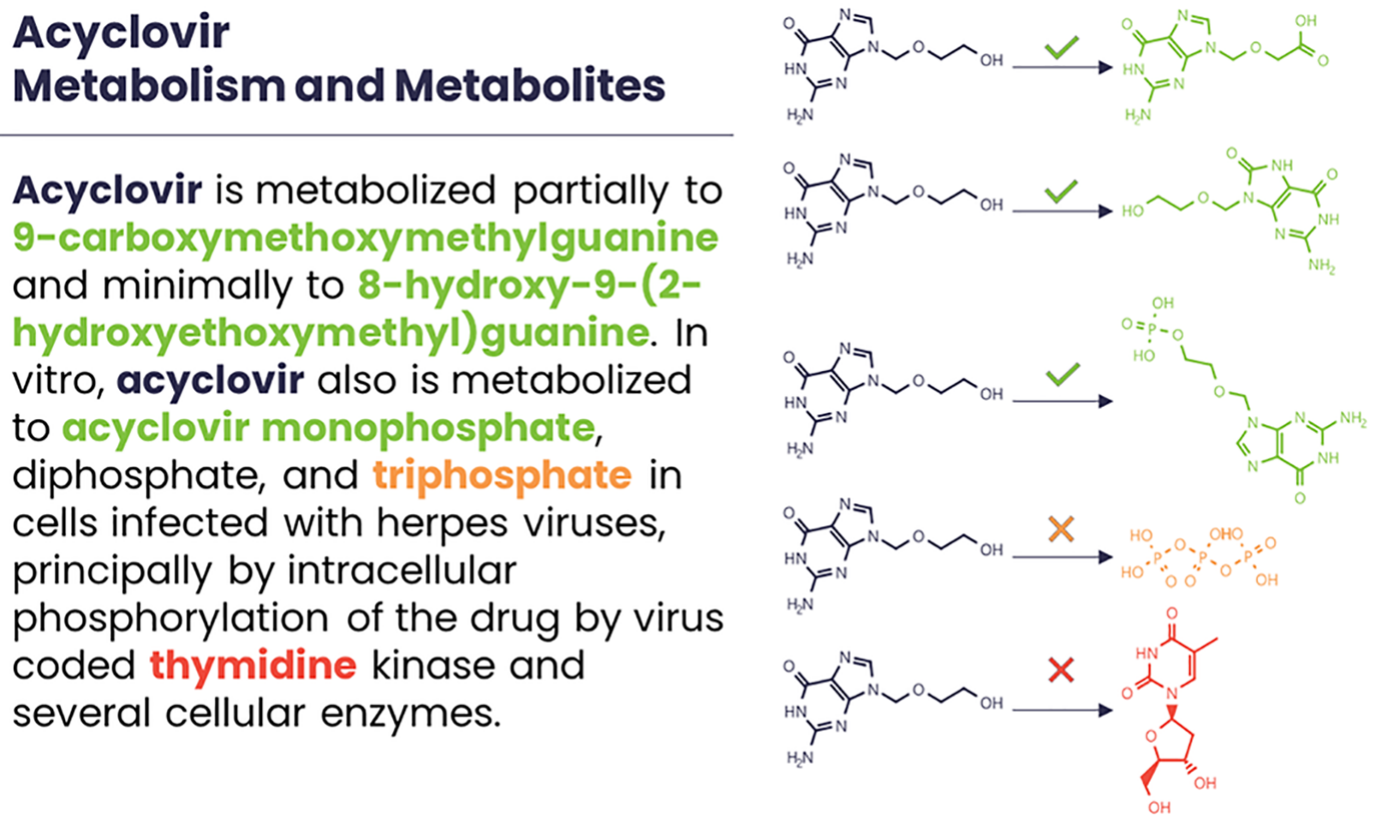
An example of the text-mined chemical names in the PubChem *Metabolism and Metabolites* section illustrating the true and false reactions. Transformation products (TPs) are highlighted in green; incompletely recognized chemical names of TPs are highlighted in orange, and correctly recognized chemical names that are not TPs are highlighted in red.

Several text mining tools for chemical name recognition have been developed to support the extraction of chemical information from text. Still, manual validation is often needed to ensure that the chemical names were recognized correctly and that no information has been omitted. Even the most accurate software packages used for text mining of chemical names report having precision and recall around 90%, indicating that without manual validation approximately 10% of the curated information would contain errors.^21^ This becomes even more complicated when using recognition of chemical names to extract potential TPs as observed by Krier et al.^22^ for the PubChem *Metabolism and Metabolites* section. This is due to the fact that even correctly recognized chemical names may be irrelevant to the transformations occurring and thus lead to incorrect reactions if not manually validated. An example of this can be seen in Figure 1.

This study introduces “ShinyTPs”, an application designed to visualize and curate text-mined chemical reactions from PubChem. Its purpose is to aid in curating TPs and their associated reactions sourced from the *Metabolism and Metabolites* section of PubChem, and thus to enable their inclusion in downstream workflows or to serve as highly curated data sets for machine learning models.

## Materials and Methods

### Data Extraction and Processing

The transformation reaction information used in this study was from the *Metabolism and Metabolites* section of PubChem with HSDB as the data source. This source was selected since it was the largest data source in the *Metabolism and Metabolites* sections and also has high reliability, as the information was manually curated over 40 years by a scientific review panel.^23^ The text excerpts are text-mined by PubChem using LeadMine and an internal synonym dictionary to extract all chemical names and link them to their PubChem compound identifiers (CIDs). The HSDB excerpts with accompanying markup and CIDs are available as a JavaScript Object Notation (JSON) file. This JSON file was processed in R to extract the chemical names, together with the CID and other information. Other chemical identifiers, such as the Simplified Molecular-Input Line-Entry System (SMILES), were added. The resulting file was exported as a .csv file together with the original text and source descriptions. The code to create this file is available on GitLab.^24^

In addition, the PubChem Transformations table is available in full on Zenodo^12^ and was used to retrieve known reactions. The Transformations library (version 0.1.4) and the HSDB *Metabolism and Metabolites* data sets include information for 6157 and 3088 compounds, respectively. The Transformations data set is being updated periodically, and the code to do this is likewise available on GitLab.^25^ HSDB was discontinued in 2019 and the underlying data set is no longer being updated, although the chemical mappings used may change over time with updates to PubChem.

### Software and Packages

ShinyTPs is an R package centered around a Shiny application for the curation of text-mined transformation reactions from the *Metabolism and Metabolites* section in PubChem.^26^ The app was developed in R^27^ (version 4.2.1). The user interface and server were constructed using shiny^28^ (a package for developing web applications) as well as the packages shinydashboard,^29^ shinyjs,^30^ shinythemes,^31^ bslib,^32^ and miniUI.^33^ The app also utilizes functions from the DT^34^ and dplyr^35^ packages to process data, as well as chemdoodle^36^ to display and draw chemical structures.

### Using ShinyTPs

ShinyTPs is used through R by downloading the package available on GitLab.^24^ The first step is to read in a file containing the names, CIDs, and SMILES of the compounds of interest, which will create a list of tables that are used inside the app. The app is then launched with a separate function in the ShinyTPs package. Full instructions with code examples and detailed screenshots are available in the app documentation found on the GitLab repository.^24^

The ShinyTPs user interface consists of an “About” page and three main tabs: “PubChem Transformations”, “Transformation reaction curation”, and “Adding missing entries”. The “PubChem Transformations” tab (screenshots available in the documentation) allows viewing and filtering of the reactions that are already available in the PubChem Transformations library, while the remaining two tabs handle the curation. “Transformation reaction curation” is used to select and save the reactions obtained from the LeadMine text mining. It displays the structures of the input compound and chemical name found in the *Metabolism and Metabolites* text as well as the original text from HSDB. This facilitates determining whether the text-mined chemical name is a TP or parent of the input compound. Each reaction is then saved in a table in the app, which can be downloaded at the end. It is also possible to include information about the biosystem, enzyme, and transformation type (e.g., hydroxylation) for the reactions if this is found in the text.

The “Adding missing entries” tab handles curation of TPs or parent compounds that were not identified by the LeadMine text mining (e.g., “acyclovir diphosphate” in Figure 1). In this case, the structure of the compound can be drawn using chemdoodle, which returns the SMILES of the drawn structure. The compound name and SMILES of the compound are then entered as text inputs to save the reaction. Information about the biosystem, enzyme, and transformation type may also be saved as described for the “Transformation reaction curation” tab described above. Further explanation and screenshots are included in the ShinyTPs documentation on the GitLab repository.^24^

### Uploading the Curated Data

**Figure 2:**
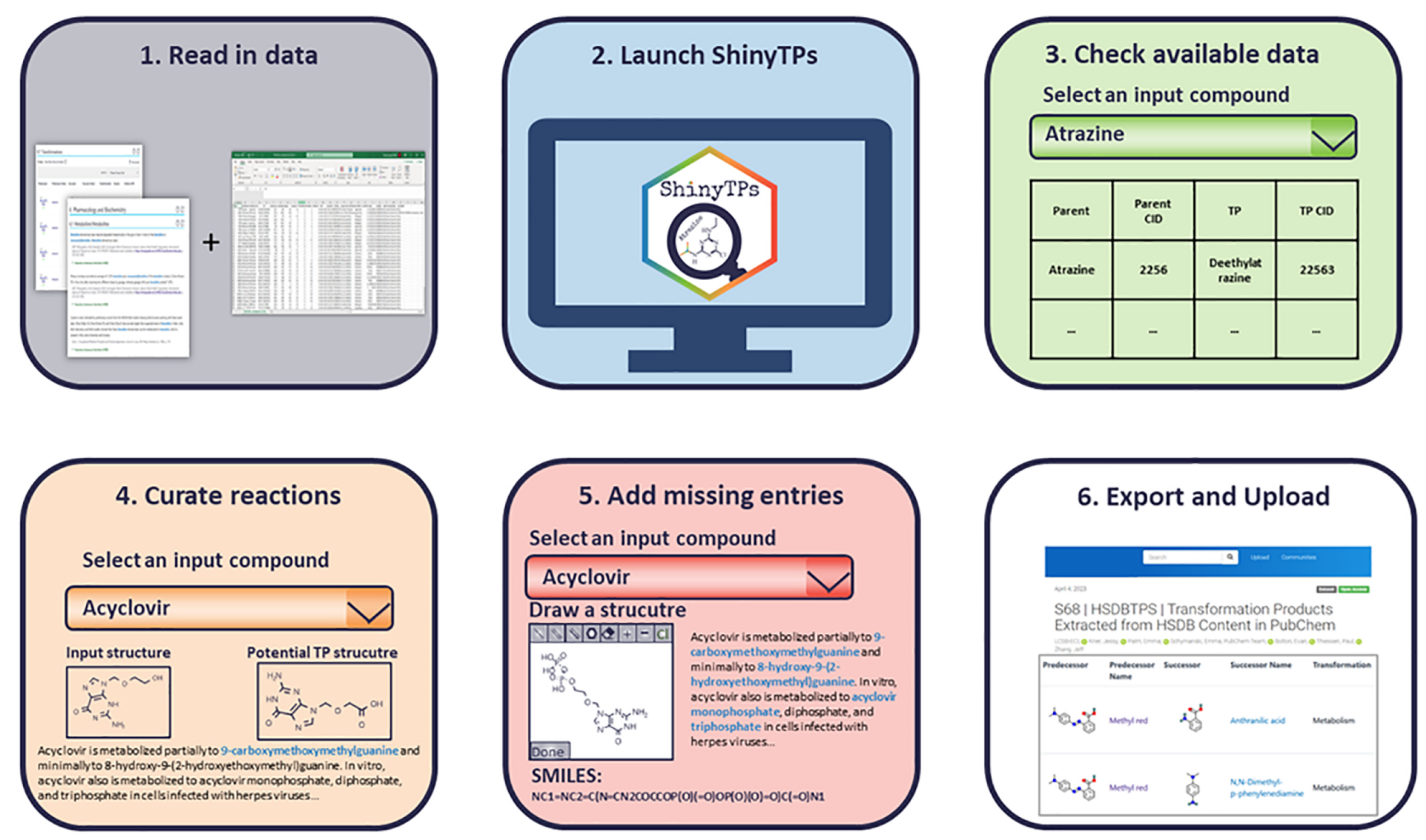
General workflow of using the ShinyTPs app to curate transformation reactions.

The output of the ShinyTPs app is designed to be included easily in the existing S68 HSDBTPS^37^ list on the NORMAN Suspect List Exchange (NORMAN-SLE).^11^ For the material prepared in this article, the information in the downloaded output files was cross-checked by the curator/creator and at least one independent reviewer using the automatically generated “reaction SMILES” via CDK Depict. Any remaining issues (e.g., salts, mismatching stereoisomers, adjustments to the descriptive information displayed) were then corrected where possible or otherwise removed from the data set. The finalized reactions were then added to the S68 HSDBTPS collection, which is integrated into the PubChem deposition and annotation cycle as part of the NORMAN-SLE efforts.^11^ Once the data was available in PubChem, the PubChem Transformations library was updated to version PubChemTrans-0.1.6.^38^ A full trace of this process is available on GitLab.^39^ An example workflow for using ShinyTPs is shown in Figure 2.

## Results and Discussion

### Application of ShinyTPs to Test Compounds

To test the application, ShinyTPs was used to curate transformation reactions for 77 compounds from five different environmentally relevant data sets with information available from HSDB in the *Metabolism and Metabolites* section using the workflow shown in Figure 2. The full list of compounds is available in Table S1 in the Supporting Information. In total, 582 chemical names were identified in the HSDB text entries in the *Metabolism and Metabolites* section. Out of those, 114 (∼20%) corresponded to valid transformation reactions where the identified chemical name was either a TP or parent compound of the compound of interest. This demonstrates why manual validation of curated reactions is very important when handling text-mined data, as there would otherwise be a significant number of incorrect reactions generated due to the presence of other chemical names in the text unrelated to transformation processes. A further 60 reactions containing chemicals that were not recognized by the text-mining tool were curated using the “adding missing entries” function based on expert knowledge. This highlights the need for not only approaches that have a higher accuracy and precision in recognizing chemical names but also the development of approaches that are able to recognize complete chemical reactions. In combination, this further demonstrates the importance of manual review and curation in combination with the text-mined entries to capture all the reactions described in the text.

Of the 174 curated reactions, 143 were not already listed in the PubChem Transformations library and 31 were listed but with different associated metadata (e.g., different biosystem, enzyme, or evidence description). In addition, 18 of the curated TP structures were not yet present in PubChem and were deposited as a part of this work (CIDs 167530376–167530379 and 168354689–168354702). This shows that much of the information available for curation in the *Metabolism and Metabolites* section of PubChem is not already available in the Transformations library, such that curating this information as done here can help fill significant knowledge gaps in the database. This may then further improve the extraction of reaction rules and the development of machine learning models for the prediction of transformation products.

**Figure 3:**
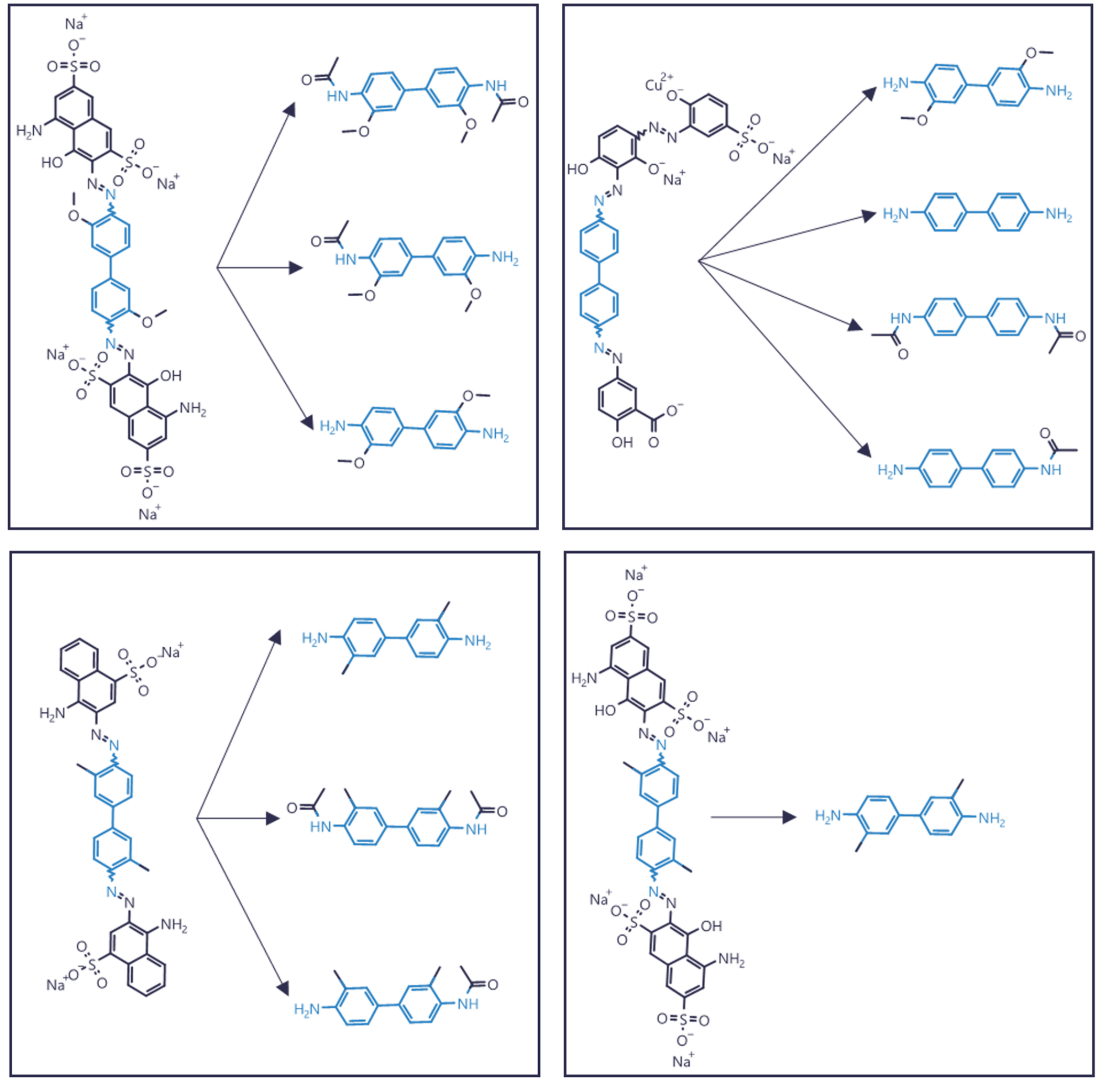
Transformation reactions of benzidine-derived azo dyes curated using ShinyTPs. The benzidine substructure is highlighted in blue. All reactions can be found on PubChem (e.g., CIDs 8413, 19074, and 626583) and Zenodo.^12,37^

Some examples of TPs curated with ShinyTPs include benzidine and congeners, formed by benzidine-derived azo-dyes, as depicted in Figure 3. Based on these reactions it was possible to derive some reaction rules such as the cleavage of the nitrogen–nitrogen double bond followed by acetylation of the amino-groups. Several of the benzidine congeners have also been found to be carcinogenic and/or mutagenic, indicating that other dyes containing the substructure highlighted in blue in Figure 3 may also form hazardous TPs.

### Application of ShinyTPs in Non-Target Workflows

**Figure 4:**
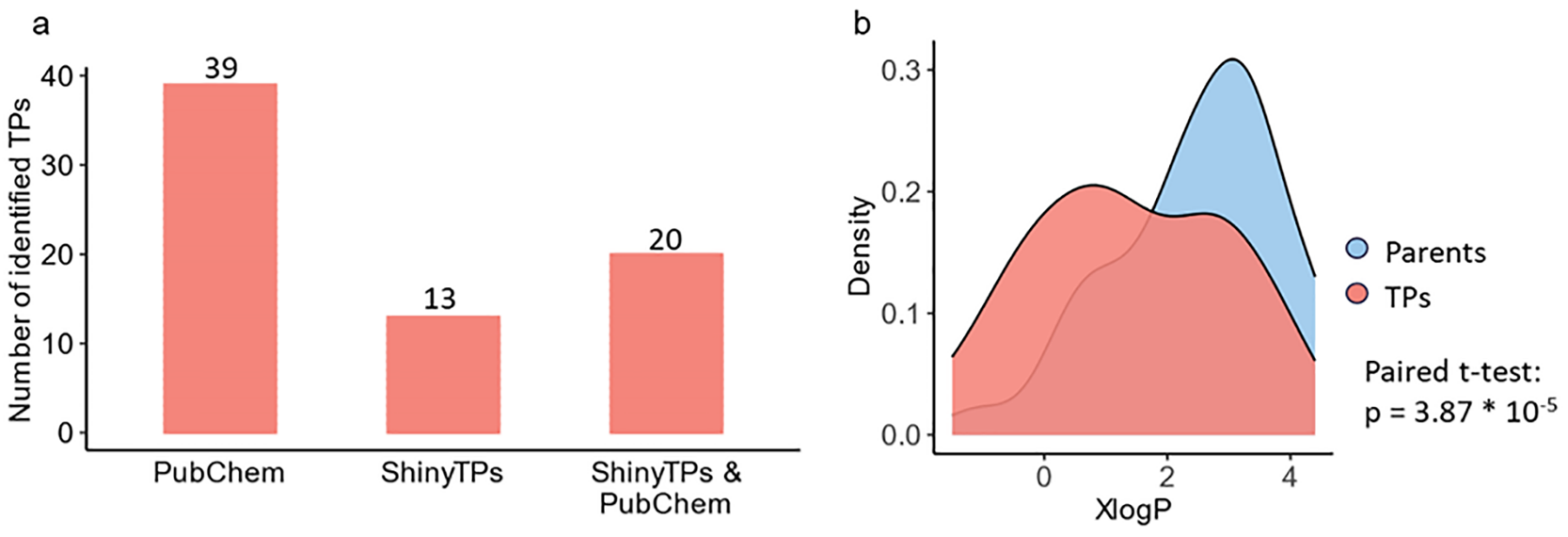
(a) Number of identified TPs only present in the PubChem Transformations library and identified TPs curated with ShinyTPs as well as the identified TPs present in both libraries. (b) Distribution of XlogP values for the identified parent compounds and their corresponding TPs. Density refers to the kernel density.^41^

ShinyTPs was also applied to curate TP information for a list of 436 tentatively identified compounds (see Table S2) from a Luxembourgish wastewater treatment plant sample. These tentatively identified compounds were used as inputs to ShinyTPs to curate associated TP information using the “Transformation reaction curation” tab, totalling 645 new reactions associated with 496 compounds. Of these reactions, 319 were not listed for any biosystem in PubChem. The PubChem Transformations library with and without the addition of the new reactions was then used to perform a TP suspect screening in patRoon with the same sample. In total, 72 compounds out of the 436 were labeled as TPs using the TP suspect lists. Out of these, 18% were detected only after curation with ShinyTPs (see Figure 4a). Thus, the additional curation of TPs using ShinyTPs contributed to the number of tentatively identified TPs in this study and demonstrates that expanding the libraries will help support non-target analysis workflows in the community.

Several of the compounds labeled as TPs did not have any identified parent compounds. This may have several causes such as degradation of the parent compound below detection limits, incorrect identification, or direct release of the compound labeled as a TP to the environment. One example of the latter is diclofenac (a common pharmaceutical observed at high intensity in wastewater), which is listed as a potential TP of aceclofenac. Excluding compounds that did not have identified parent compounds resulted in 36 parent and TP pairs. Among these, the TPs were found to have a significantly lower octanol–water partitioning coefficient (logP), via the XlogP^40^ values calculated in PubChem, compared to their parent compounds (see Figure 4b). This in turn suggests a higher mobility of TPs compared with their parents. Some TPs were also associated with several parent compounds, e.g., terbuthylazine-2-hydroxy (see Table S3), which is a TP of terbuthylazine and terbutryn. This may be an indication of a higher potential for accumulation of these TPs, especially for those with longer half-lives, such as triazines. All tentatively identified TPs and their corresponding parents can be found in Table S3.

### Future Perspectives

User contributions will help expand open transformation product libraries, which in turn can help improve screening of environmental contaminants, as illustrated by the application in this article. As the use of ShinyTPs expands (already external users are using the application), it is important to avoid curating duplicate information. This increases the importance of utilizing the “PubChem Transformations” tab to see which reactions are already available. Since HSDB was discontinued in 2019, new compounds will not be added to the HSDB data set and thus the TPs of emerging contaminants are not likely to be present. However, it will also be possible to enhance ShinyTPs to other sections of PubChem (e.g., the “Pharmacodynamics” section) in the future to expand the scope of this effort further. Finally, for current studies describing transformation processes, the inclusion of the results in a standard FAIR reporting template could ease the addition of these TP reactions into public resources.^42,43^

## Supporting Information Available

Tables S1–S3 containing lists of compounds used in the initial evaluation of ShinyTPs (S1), plus compounds (S2) and TPs (S3) tentatively identified in the wastewater treatment sample (XLSX)

## Notes

Associated Content: see ref. (44).
